# Human papillomavirus prophylactic vaccines: update on new vaccine development and implications for single-dose policy

**DOI:** 10.1093/jncimonographs/lgae026

**Published:** 2024-11-12

**Authors:** Anne E Schuind, Kanduri Ananth Balaji, Anna Du, Yuan Yuan, Peter Dull

**Affiliations:** PATH, Center for Vaccine Innovation and Access, Washington, DC, USA; PATH, Center for Vaccine Innovation and Access, New Delhi, India; Bill & Melinda Gates Foundation, Beijing, China; PATH, Center for Vaccine Innovation and Access, Shanghai, China; Bill & Melinda Gates Foundation, Seattle, USA

## Abstract

Human papillomavirus (HPV) prophylactic vaccines were first licensed in 2006 with the primary goal of preventing HPV-related cancers, with cervical cancer accounting for the highest morbidity and mortality globally. Six HPV vaccines have been licensed; 4 of these have been prequalified by the World Health Organization, and additional products are in the pipeline. This article provides an overview of HPV vaccine coverage and current and anticipated vaccine supply vs expected demand. Given that the 2022 World Health Organization position paper on HPV vaccines includes a 1-dose regimen as an alternate schedule, we will discuss the evidence for using licensed vaccines in single-dose regimens and the approach to generating similar supportive data for other current and future vaccines. The broad adoption of a single-dose HPV vaccine regimen would expand access to vaccines by improving the supply-demand balance, increasing affordability, and simplifying logistics, which will ultimately impact HPV-related morbidity and mortality.

Globally, the burden of human papillomavirus (HPV)–related malignancies, including cancers of the cervix, anogenital tract, and head and neck, represents 4.5% of new cancer cases (1). Cervical cancer, accounting for 83% of the total burden of cancer attributable to HPV, is the main focus of this manuscript. Cervical cancer remains a disease of geographic and economic inequity, being the second most common cancer in women in low-income countries (2). Regional differences in the cervical cancer burden are related to disparities in access to vaccination, screening, and treatment services as well as risk factors such as HIV prevalence and social and economic determinants. The World Health Organization (WHO) International Agency for Research on Cancer estimated that in 2022, there were more than 660 000 new cases of cervical cancer and nearly 350 000 cervical cancer–related deaths, with approximately 84% of new cases and 90% of deaths occurring in low- and middle-income countries (LMICs) (2).

Persistent infection with HPV is a necessary precursor to cervical cancer (3). Cervical cancer is a preventable disease. Primary prevention via prophylactic HPV vaccination is safe and highly effective (4) and thus has tremendous potential to save lives, particularly in countries where secondary prevention through cervical cancer screening and treatment may not be accessible. Despite more than 16 years of vaccine availability, only 1 in 8 girls globally was vaccinated against HPV in 2023 (5).

In 2018, the WHO director general called for global action to eliminate cervical cancer, defined as an age-standardized incidence rate of or below 4 per 100 000 women. The WHO Cervical Cancer Elimination Initiative outlines in its strategy a set of targets to achieve by 2030: 90% of girls vaccinated by age 15 years, 70% of women screened with high-performance tests by age 35 years and again by age 45 years, and 90% of women identified with cervical disease treated. Achieving these targets in every country could reduce cervical cancer incidence rates by 97% and more than 74 million cervical cancer cases and 62 million deaths prevented by 2120 (6). Meeting the WHO goals by 2030 will require countries to intensify and accelerate their cervical cancer elimination strategies.

## HPV vaccine access, delivery, and funding

As of April 2024, 137 WHO member states have included HPV vaccination in their national vaccination programs, and 54 countries have adopted a gender-neutral (ie, vaccination of girls and boys) vaccination strategy (7). In 2020, less than 30% of LMICs had introduced HPV vaccination in their national immunization programs (6) in contrast to more than 85% of high-income countries. Despite the steady progress in countries introducing HPV vaccination, 57% of global cervical cancer cases occur in countries that do not yet have an HPV vaccination program in place (8).

The COVID-19 pandemic substantially impacted HPV vaccination coverage because of supply issues, school closures, and health system disruptions. In 2023, despite some recovery, the global coverage remains low at 21% in females and 7% in males by age 15 years (9), which is far below the WHO cervical cancer elimination target. There is much variability in global coverage among WHO regions (10), from 3% in the Southeast Asian region to 76% in the region of the Americas.

In the context of immunization programs and compared with other Expanded Program on Immunization vaccines, the costs associated with delivery of HPV vaccines are high, because the target population are pre-adolescents and adolescents, consequently stretching already limited health budgets and representing a major hurdle to scalability and sustainability. In addition, delivery of HPV vaccine in a multidose schedule represents a considerable operational hurdle for most countries. Global, regional, and local organizations are partnering with countries to provide resources to address these challenges.

By allowing for predictable funding and aggregating demand, Gavi, the Vaccine Alliance, has created a model that allows vaccine manufacturers to adopt a tiered pricing policy, whereby low-income countries are charged less than higher-income countries for the same product (11). Eligibility for Gavi support is based on a country’s gross national income threshold, and requests must be initiated by the countries. UNICEF may be able to secure improved pricing for middle-income countries from manufacturers through long-term procurement commitments based on identified long-term HPV vaccine needs. In a similar way, the Pan American Health Organization Revolving Fund provides a mechanism for Gavi-ineligible Latin American and Caribbean countries to access HPV vaccines at a reduced price through pooled procurement and discounted pricing strategies.

The impact of vaccine costs is considerable for countries transitioning out of Gavi support and for self-procuring countries. Not being able to access negotiated vaccine prices represents an affordability barrier for these countries (12). To successfully eliminate cervical cancer, focused alignment among governments, global partners, and suppliers will be needed.

## HPV vaccine supply and demand

### Supply through Gavi

Following an increase in demand for HPV vaccines in 2016, Gavi’s HPV vaccine program experienced a major supply-demand imbalance. This was further exacerbated by increased global interest in response to WHO’s call for action toward global cervical cancer elimination as well as increased adoption of gender-neutral policies, the latter mostly in high-income countries. With an increase in the manufacturing capacities of existing suppliers and the entry of new suppliers into the market, the supply constraints are easing, with aggregate supply expected to meet demand in 2025 for Gavi-eligible countries (13). Gavi forecasts demand of 45-55 million doses annually until approximately 2027 with the demand stabilizing around 35 million doses thereafter. This forecast takes into consideration that the program covers the WHO-recommended primary target population of girls aged 9-14 years as well as girls through age 18 years for countries with delayed multi-age cohort vaccinations adopting a 1-dose schedule. It further assumes that most countries with existing HPV vaccine programs will have switched to a 1-dose schedule and that countries introducing HPV vaccine into their national immunization programs will adopt a 1-dose schedule by the end of 2025.

### Global supply

In 2020, the global market demand for HPV vaccines was projected to reach approximately 44.5 million doses, with LMICs and low-income countries accounting for 18% and 12% of the global market, respectively (14). The global demand for HPV vaccines is anticipated to increase substantially to 140 million doses per year in 2026, because of the introduction in China and India and the adoption of gender-neutral vaccination policies. The demand is expected to stabilize around 125 million doses per year after 2030 following completion of multi-age cohorts (15).

The 2 most populated countries in the world, India and China, have yet to introduce HPV vaccines into their national immunization programs, despite the fact that several HPV vaccines have obtained local licensure. This is presumably in part because of the ability to guarantee a reliable supply of sufficient doses of vaccines at an affordable price. The entry of locally manufactured HPV vaccines into domestic and global markets is likely to substantially increase the worldwide availability of lower-cost HPV vaccines (16,17).

In China, each year of delay in initiating a large-scale vaccination program could result in an estimated 119 000 cervical cancer cases and 42 000 deaths over the lifetime of women and more than 1 year of delay to achieving cervical cancer elimination (18). According to the estimation made by the National Cancer Center of China, approximately 80 million doses of HPV vaccines are needed to vaccinate 90% of girls aged 9-14 years (19). Since 2020, China has expedited a series of efforts promoting free HPV vaccination pilot programs and launching action plans to scale up HPV vaccination. As of September 2023, of the 32 provincial-level administrative regions in the mainland of China, 7 had implemented free provincial HPV immunization programs in girls aged 13 or 14 years (18).

Cervical cancer is a major public health problem in India, contributing nearly one-fifth of the global burden with an estimated 123 907 new cases and 77 348 deaths in 2020. India has the largest adolescent population in the world, estimated in 2022 to include approximately 120.4 million women aged 10-19 years (20). In June 2022 and July 2023, the National Technical Advisory Group on Immunization recommended the introduction of HPV vaccine in the Universal Immunization Program of India with the indigenously developed quadrivalent HPV vaccine as a 2-dose regimen for adolescent girls aged 9-14 years (21). According to a report in *The Lancet Oncology*, over the subsequent 3 years (2024-2026), the Indian Ministry of Health and Family Welfare may opt for a phased rollout of HPV vaccination targeting 68 million girls aged 9-14 years; subsequently, each year, 11.2 million girls aged 9 years could be targeted for the routine HPV vaccination program in India (17). One modeling study estimated that HPV vaccination in India would prevent approximately 1 million cases of cervical cancer over the lifetime of birth cohorts currently aged 10 years or younger. Further, adopting a single-dose vaccination schedule could free up resources that could be used to extend vaccination beyond the primary target of girls aged 9-14 years to those aged 15-20 years, preventing an additional 800 000 cases of cervical cancer (22).

## L1 virus-like particles-based HPV vaccines

### Currently licensed vaccines

As of April 2024, there are 6 licensed prophylactic HPV vaccines, all based on the L1 major capsid antigen that self-assembles into virus-like particles: 3 bivalent vaccines against oncogenic types HPV-16 and -18 (Cervarix [GSK], Cecolin [Innovax], Walrinvax [Walvax/Zerun]); 2 quadrivalent vaccines against low-risk types HPV-6 and -11, in addition to HPV-16 and -18 (Gardasil [MSD] and Cervavac [Serum Institute of India]); and 1 nonavalent vaccine against 5 additional high-risk types HPV-31, -33, -45, -52, and -58 (Gardasil9 [MSD]). Of those, Cervarix, Cecolin, Gardasil, and Gardasil9 have obtained WHO prequalification (23), which allows procurement through agencies such as UNICEF. HPV vaccines currently available for procurement through UNICEF are Cervarix, Gardasil, and Cecolin. Several suppliers utilize bulk transfer to fill products for local distribution and market the finished vaccine under a domestic name (Incepta, Bangladesh markets Papilovax from Innovax; Biofarma, Indonesia markets NusaGard from MSD). MSD is also commercializing its HPV vaccines with 2 local market authorization holders: Instituto Butantan in Brazil and Sinergium Biotech in Argentina. Despite agreements to perform full technology transfer for drug substance manufacturing, these steps have not happened yet for any HPV vaccines (15).

### Review of products in development

As has occurred with other vaccines, the entry of HPV vaccine from new manufacturers into domestic and global markets has the potential to substantially increase the availability of lower-cost HPV vaccines worldwide. It is important not only for ensuring sufficient supply but also for ensuring affordable vaccine pricing by mitigating the price-setting power of well-established manufacturers (24).

Table 1 shows the L1 virus-like particle–based HPV vaccines currently in clinical development, mainly in China but also in other parts of Asia, with their composition as well as their stage of development.

**Table 1.** L1 virus-like particle–based human papillomavirus vaccines: products in clinical development

Manufacturer	Antigens	Expression system	Adjuvant	Research and development phase	Populations, years of age, and schedule^a^
China					
Bovax	6, 11, 16, 18	Yeast (*H polymorpha*)	Aluminum phosphate	Phase 3	Females, 9-45 3 doses (0, 2, 6 mo)
Bovax	6, 11, 16, 18, 31, 33, 45, 52, 58	Yeast (*H polymorpha*)	Aluminum phosphate	Phase 3	Females, 9-45 Males, 9-45 3 doses (0, 2, 6 mo)
CDBIO/Health Guard	6, 11, 16, 18, 31, 33, 35, 39, 45, 51, 52, 56, 58, 59, 68	*E coli*	Not disclosed	Phase 1	Females 9-45 3 doses (0,2,6 mo)
CNBG/CDIBP	6, 11, 16, 18	Yeast (*H polymorpha*)	Aluminum based	Phase 3	Females, 18-45 3 doses (0, 2, 6 mo)
CNBG/CDIBP	6, 11, 16, 18, 31, 33, 45, 52, 58, 59, 68	Yeast (*H polymorpha*)	Not disclosed	Phase 3	Females, 18-45 3 doses (0, 2, 6 mo)
CNBG/SIBP	16, 18, 52, 58	Yeast (*P pastoris*)	Aluminum hydroxide	Phase 2	Females, 9-45 3 doses (0, 2, 6 mo)
Health Guard	16, 18, 58	*E coli*	Aluminum based	Phase 3	Females, 9-45 3 doses (0, 1, 6 mo)
Health Guard	6, 11, 16, 18, 31, 33, 45, 52, 58	*E coli*	Aluminum hydroxide	Phase 3	Females, 9-45 Males, 18-45 3 doses (0, 2, 6 mo)
Immune Path/Ab&B bio	6, 11, 16, 18, 31, 33, 45, 52, 58	Yeast (*P pastoris*)	Novel adjuvant	Phase 2	Females, 18-45 2 doses (0, 2 months; 0, 6 mo)
Innovax	6, 11	*E coli*	Aluminum based	Phase 2	Females, 18-55 3 doses (0, 1, 6 mo)
Innovax	6, 11, 16, 18, 31, 33, 45, 52, 58	*E coli*	Aluminum based	Phase 3	Females, 9-45 3 doses (0, 1, 6 mo)
Nuoning/Sinocelltech	6, 11, 16, 18, 31, 33, 35, 39, 45, 51, 52, 56, 58, 59	Insect cell	Aluminum based	Phase 3	Females, 18-45 3 doses (0, 2, 6 mo)
RecBio	16, 18	Yeast (*H polymorpha*)	Aluminum based	Phase 1	Females, 9-45 3 doses (0, 2, 6 mo)
RecBio	6, 11	Yeast (*H polymorpha*)	Aluminum based	Phase 1	Females, 18-45 3 doses (0, 2, 6 mo)
RecBio	6, 11, 16, 18, 31, 33, 45, 52, 58	Yeast (*H polymorpha*)	Aluminum based	Phase 3	Females, 9-45 3 doses (0, 2, 6 mo)
Zerun/Walvax	6, 11, 16, 18, 31, 33, 45, 52, 58	Yeast (*P pastoris*)	Aluminum phosphate	Phase 3	Females, 9-45 3 doses (0, 2, 6 mo)
India and other Asian countries					
SK Bioscience	6, 11, 16, 18	Not disclosed	Not disclosed	Phase 1 and 2	Females 9-26
Other countries					
GlaxoSmithKline	Nonavalent vaccine	*E coli*	AS04	Phase 2	Females, 16-26

a Information from clinical trial registries. *E coli* = *Escherichia coli*; *H polymorpha* = *Hansenula polymorpha*; *P pastoris* = *Pichia pastoris*.

A search of clinical trial registries shows that there are currently more than 18 products in clinical development with at least 10 having initiated phase 3 studies to support licensure. Most of the products are being developed in China and thus require large and lengthy studies with clinical endpoints to meet local regulatory requirements. It is notable that half of the candidate vaccines includes 9 or more HPV types and thus increases the likelihood for an expanded supply of second-generation vaccines. Several manufacturers have also included males in their clinical development, with the prospect to obtain an indication for both genders.

In addition to products shown in Table 1, multiple companies have indicated an interest to advance higher valency L1-based vaccines into clinical development. There have also been preclinical studies looking at adding proteins (eg, L2) or enhancing cross-reactivity to multiple L1 targets, but none have yet entered clinical development.

## Pathways for use as single-dose vaccines

### Pathway to licensure for HPV vaccines

Licensure of HPV vaccines was originally granted in young adult women for a 3-dose regimen based on the demonstration of efficacy in preventing cervical precancer lesions related to the types targeted by the vaccines. A surrogate endpoint for cervical cancer was used for ethical and practical reasons. Therefore, initial licensure of GSK and MSD vaccines evaluated the prevention of high-grade cervical dysplasia, which required large randomized controlled trials with a follow-up of approximately 4 years to enable accrual of sufficient cases in a population of sexually active women who were HPV seronegative and HPV DNA negative prior to vaccination (25). Vaccine efficacy against persistent infections (ie, the detection of the same HPV type in consecutive cervical specimens taken several months apart) was a secondary objective in these studies. High efficacy was compatible when measured against either persistent infections or high-grade cervical lesions.

The indication was extended to girls beginning at 9 years of age using the principle of immunobridging. Because demonstration of efficacy in a young population presents ethical and practical hurdles and would require large trials of extended duration, effectiveness in pre-adolescent and adolescent populations was inferred based on the demonstration of comparable immune responses 1 month following the completion of the dosing regimen, using as benchmark the immune response elicited in an adult population for which clinical efficacy has been demonstrated (26,27).

In 2013, an experts workshop convened by the International Agency for Research on Cancer and the US National Cancer Institute recommended the use of prevention of persistent HPV infections as a primary efficacy endpoint, as infections occur more frequently than cervical dysplasia, and this endpoint is more reproducible. Immunological noninferiority was also found as an acceptable endpoint for licensure of alternate dosing schedules, new populations, second-generation vaccines (for shared HPV types), and biosimilar vaccines (ie, L1 virus-like particle–based HPV vaccines) (25). This recommendation was subsequently adopted by WHO (28).

The higher responses in children compared with adults led to the exploration of a 2-dose regimen on a 0, 6-month schedule compared with the 3-dose regimen (29,30). Based on the outcome of these immunobridging studies, regulatory agencies and WHO updated their guidance in 2014 for reduced schedule of 2 doses for girls aged 9-14 years, while a 3-dose schedule remained indicated for women aged 15 years and older as well as for immunocompromised individuals (31,32).

Despite the recommendation from WHO to consider alternate endpoints for pivotal HPV trials, authorities in several countries, including China, still require, for more recently developed HPV vaccines, the demonstration of efficacy based on prevention of high-grade cervical lesions in an adult population (33). The Serum Institute of India quadrivalent HPV vaccine is currently the first vaccine to have obtained regulatory approval in a population aged 9-26 years solely on the basis of an immunogenicity assessment comparable with a licensed quadrivalent vaccine with demonstrated efficacy (16). The indication in the younger age population was obtained through demonstration of immunological noninferiority between the candidate vaccine in girls aged 9-14 years vs the active comparator vaccine in women aged 15-26 years (34).

All licensed vaccines are currently indicated on a 3-dose regimen for individuals aged older than 15 years or for individuals with immunocompromising conditions, whereby in pre-adolescents and adolescents, a 2-dose schedule is available.

### Recommendation for HPV single-dose vaccination

Over the last decade, vaccine trials and real-world evidence evaluations have provided increased confidence in high and durable effectiveness of a single-dose schedule against HPV infection compared with multidose schedules (35-37). Despite lower antibody levels elicited by a 1-dose compared with 2- or 3-dose regimens, the immune response is sustained for at least 10 or more years postvaccination (38,39). More recently in a randomized clinical trial vs delayed vaccination, single-dose HPV vaccination provided at least 98% efficacy against persistent HPV infections persisting through at least 3 years (40). Effectiveness of a single-dose regimen was further demonstrated through immunobridging to support its use in a population of pre-adolescents and adolescents (41,42).

Based on the body of evidence related to HPV single-dose vaccination and following the advice of the WHO Strategic Advisory Group of Experts on Immunization, in December 2022, WHO revised its position paper for HPV vaccination to include a 1-dose schedule as an option for HPV vaccination in girls and boys aged 9-20 years (4).

**Figure 1.**
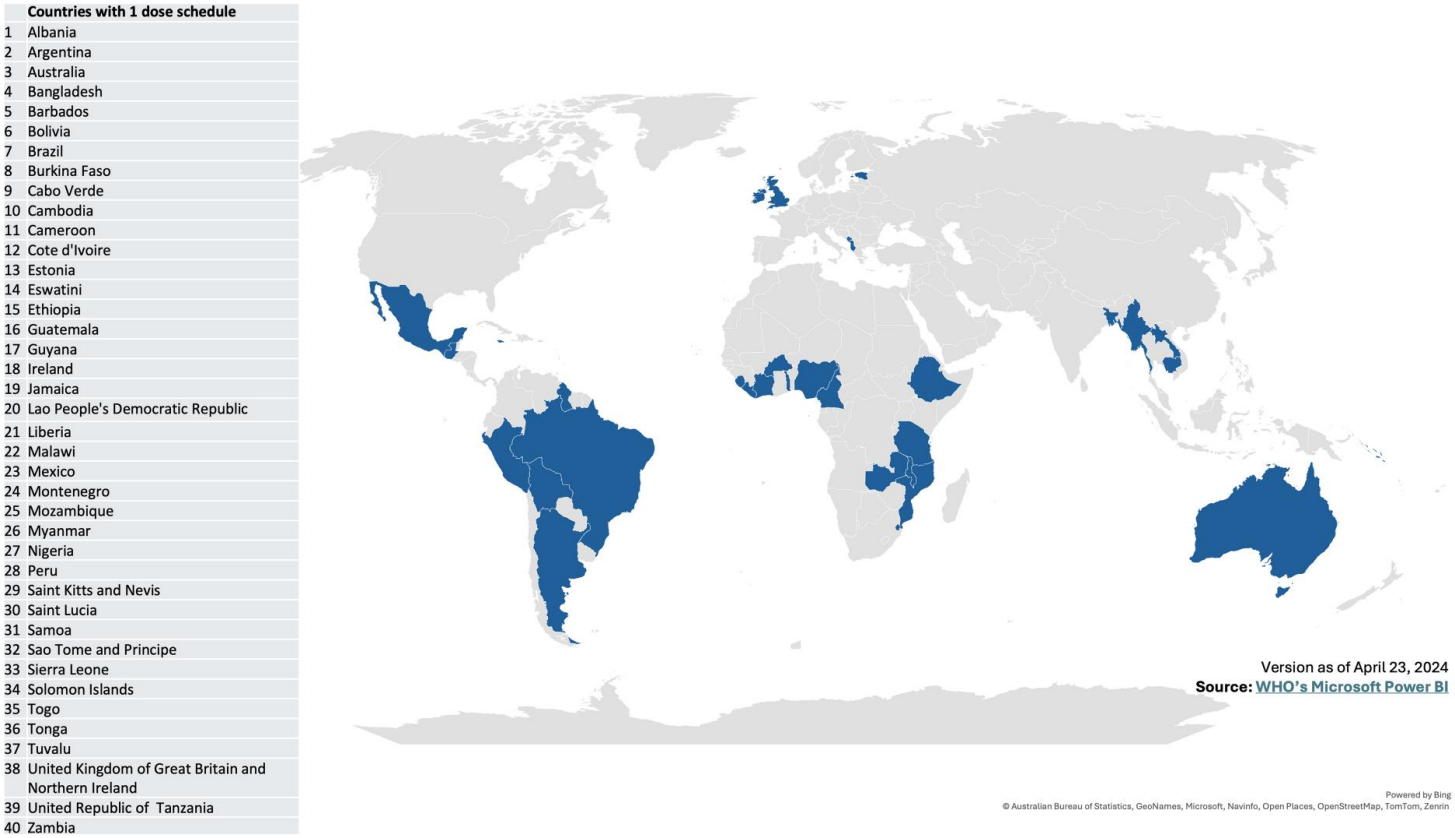
Countries having adopted a single-dose human papillomavirus vaccine schedule as of April 23, 2024.

Several high-income countries and LMICs are adopting single-dose HPV vaccination into their national immunization programs either by switching from a multidose to a single-dose regimen or introducing HPV vaccination directly on a single-dose schedule. As of April 2024, a total of 40 countries have included a single-dose HPV vaccination strategy in their immunization programs, and additional countries are planning introductions or switches to a single-dose regimen (see Figure 1).

### Pathway from single-dose recommendation to single-dose indication

The alternative single-dose schedule endorsed by WHO in 2022 covers vaccination of females and males aged 9-20 years (4). This schedule has not been evaluated by regulatory agencies and is therefore considered off-label use. Nevertheless, WHO endorsed a single-dose schedule because of compelling evidence for high and durable efficacy comparable with multidose regimens and the potential for important programmatic advantages. Thus, from a public health perspective, single-dose HPV vaccination benefit is thought to outweigh a potential risk of a lower level of protection should efficacy wane over time. This option does not apply to immunocompromised persons including those living with HIV, who should receive at least 2 HPV vaccine doses and, when possible, 3 doses (4).

Noteworthy, the alternative single-dose recommendation applies only to vaccines that have either efficacy or immunobridging data supporting a single HPV vaccination. This is currently the case for 3 vaccines: GSK bivalent (Cervarix), MSD quadrivalent (Gardasil), and MSD nonavalent (Gardasil9) vaccines. For vaccines licensed recently or in the future, the WHO position paper specifies that single-dose regimens will need to be evaluated in clinical trials to receive a similar recommendation (4). That is, either new single-dose efficacy will need to be demonstrated or immunobridging performed (comparison at peak and plateau [24 months]) to one of the vaccines for which single-dose efficacy has been supported.

As of today, none of the licensed HPV vaccines is indicated for single-dose administration. A label change would require the marketing authorization holder to submit supportive data for evaluation by a regulatory authority. There are different approaches to generating primary single-dose efficacy as demonstrated by recent studies either performed [36] or ongoing [Costa Rica ESCUDDO trial (43) and PRISMA trial (44)]. Each of these have context-specific considerations for their design that relate to identification of an appropriate control group. Creative alternative designs can also be considered, including the use of nonvaccine strains to estimate attack rates (45). It may also be the case that a label claim could be proposed based on an immunobridging approach similar to that recommended in the WHO position paper (28). In a press release dated March 13, 2024, MSD announced plans to conduct 2 separate clinical trials in females and males aged 16-26 years to evaluate the efficacy and safety of a single-dose regimen compared with a 3-dose regimen of Gardasil9 (46).

Ultimately, it will be the responsibility of a vaccine marketing authorization holder to submit a data package sufficient for regulatory authorities to grant a single-dose indication. This approach was adopted nearly 10 years ago for the 2-dose reduced schedule that is now universally applied for young populations.

## Discussion

HPV prophylactic vaccination, the primary prevention measure for cervical cancer, is 1 of the 3 pillars of the cervical cancer elimination strategy articulated by WHO. Despite HPV vaccines having first been licensed more than 15 years ago, the global coverage remains low, particularly in LMICs where the cervical cancer disease burden is highest. Although there are multiple licensed HPV vaccines, global access is limited by high cost and uneven distribution, and there is a need for additional products. There is a healthy pipeline of new vaccines in late-stage development, including high-valency products, but broad availability requires WHO prequalification. Although those products are currently being evaluated in multidose schedules, the scientific evidence warrants these vaccines to be also evaluated in a single-dose regimen. A 1-dose vaccination regimen would improve the supply-demand balance and address many challenges faced by countries with respect to HPV vaccination. Therefore, single-dose HPV vaccination has the prospect to improve vaccine access by increasing vaccine uptake or expanding HPV vaccination to countries currently unable to afford it, as well as by expanding vaccination to secondary target populations. This is a unique opportunity for communities, policy makers, regulators, and vaccine manufacturers to contribute to the cervical cancer elimination effort.

## Data Availability

No new data were generated or analysed in support of this manuscript.
